# Radioactivity and radiological hazards from a kaolin mining field in Ifonyintedo, Nigeria

**DOI:** 10.1016/j.mex.2018.04.009

**Published:** 2018-04-16

**Authors:** T.A. Adagunodo, A.I. George, I.A. Ojoawo, K. Ojesanmi, R. Ravisankar

**Affiliations:** a Department of Physics, Covenant University, Ota, Ogun State, Nigeria; b Department of Physics, University of Ibadan, Ibadan, Oyo State, Nigeria; c Department of Chemistry, Covenant University, Ota, Ogun State, Nigeria; d Post Graduate and Research Department of Physics, Government Arts College, Tiruvanamalai 606603, Tamilnadu, India

**Keywords:** Kaolin, Ceramic raw materials, Ifonyintedo, Dahomey Basin, Radioactivity, Radiological hazards, Miners

## Abstract

The concentrations of the radionuclides in the subsurface formation (soils and rocks) solely depend on their geological origin, which enables its variation from point to point on the Crust. Construction materials can possess elevated concentrations of radioactivity if their byproducts are mined from contaminated radionuclide sources. In this article, results of in situ measurements of radioactivity concentrations of ^40^K, ^232^Th, and ^238^U as well as gamma doses and radiological hazards from kaolin mining field were presented and evaluated. Eleven stations were randomly occupied in order to cover the upper axis of a kaolin mining field in Ifonyintedo. The radiometric survey was achieved using Super-Spec (RS-125), equipment capable of measuring activity concentrations and gamma doses. For each location, measurements were taken four times, while its mean and standard deviation values were estimated for better accuracy. The overall mean activity concentrations (for ^40^K, ^232^Th and ^238^U) and gamma dose were estimated as 93.9 Bq kg^−1^, 65.1 Bq kg^−1^, 38.2 Bq kg^−1^, and 59.6 nGyh^−1^ respectively. The estimated radiological hazards from the measured parameters showed that the overall mean concentrations of Radium Equivalent, External and Internal Hazards, Annual Effective Dose, Gamma and Alpha Indices, and Representative Level index are 138.5 Bq kg^−1^, 0.37 0.48, 0.29 mSvyr^−1^, 0.48, 0.19, and 0.97 respectively. By comparing the mean values of the activity concentrations and their radiological risks with the several world standards from the literature, kaolin deposits in Ifonyintedo are highly rich in thorium.

## Highlights

• The in situ measurements of radioactivity concentrations and gamma doses from a kaolin mining field were presented.
• The estimated radiological hazards showed some hazardous locations in the study area.
• Kaolin deposits in Ifonyintedo, Nigeria are highly rich in thorium.

## Graphical abstract

**Figure:**
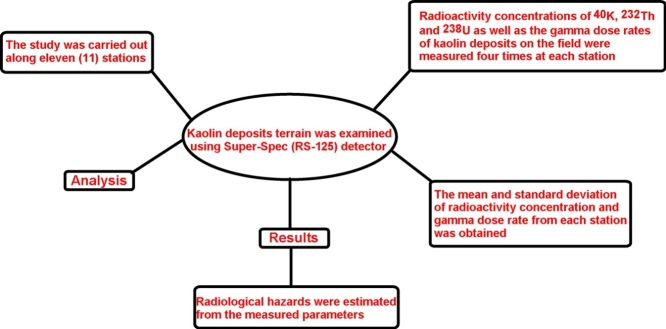

## Method details

Kaolin is one of the types of clay found in nature, with the chemical composition of Al_2_Si_2_O_5_(OH)_4_ [1]. The name “kaolin” is derived from a Chinese word Gaoling, which literally mean “High Ridge”. The industrial usefulness of kaolinite clays can be found in paper industry [2], paint industry (as filler for paint), rubber and plastic industry [3], and construction industry [4]. They are used in the production of ceramics, cement, porcelain and bricks [5], toothpaste, food additive, and cosmetics [6]. Kaolinite clay also found its application in agricultural domain (production of spray that repel insects and avert sun burn) and medicine [6]. Recent study from Turkey showed that Kaolin clays are cost effect when used as pozzolanic additives in cement and concrete [7].

Ceramic raw materials are categorized into plastic and non-plastic ceramics. The former are materials that exhibit plasticity property when mixed with water, which include kaoline, bentonite and clay. The later are materials that are not plastic when mixed with water, which include feldspar, quartz, dolomite, limestone, magnesite, talc and calcium phosphate [8]. The main component in ceramic tile body is clay. Clay is a term for naturally occurring mineral aggregates consisting mainly of the hydrous silicate of alumina. Tile is a thin rectangular or square slab of baked clay used in overlapping rows for covering floor, wall column, or and roof. Geologically, kaolin is a result produced when feldspar crystals and feldspar are mixed together under the control of weathering [9]. As stated on the website of Ceramic Research Company [8], weathering is a wearing down of all exposed rock body that is frequently breaking down to sea level by actions such as water, glacial or wind. It is either a mechanical or physical process.

Globally, elevation of activity concentrations of radionuclide and its radiological consequences from buildings as well as building materials from geological origin have been reported by many researchers, among are: Lu et al. [10], Arabi et al. [11], Ge and Zhang [12], and Isinkaye et al. [13]. Recently, activity assessment and radiological risks associated with tiles made in Nigeria have been reported by Joel et al. [14] and Joel et al. [15]. Their results showed elevated concentrations of radionuclides in different tiles manufactured in Nigeria. Their outcome has facilitated this research in order to evaluate the radionuclide concentrations of one of the major kaolin deposits used for manufacturing of tiles in Nigeria. However, the aim of this research is to assess the concentrations of radioactivity on a kaolin mining field in Ifonyintedo, Nigeria and to estimate the radiological risks to human exposure.

## The study area and its geology

**Fig. 1:**
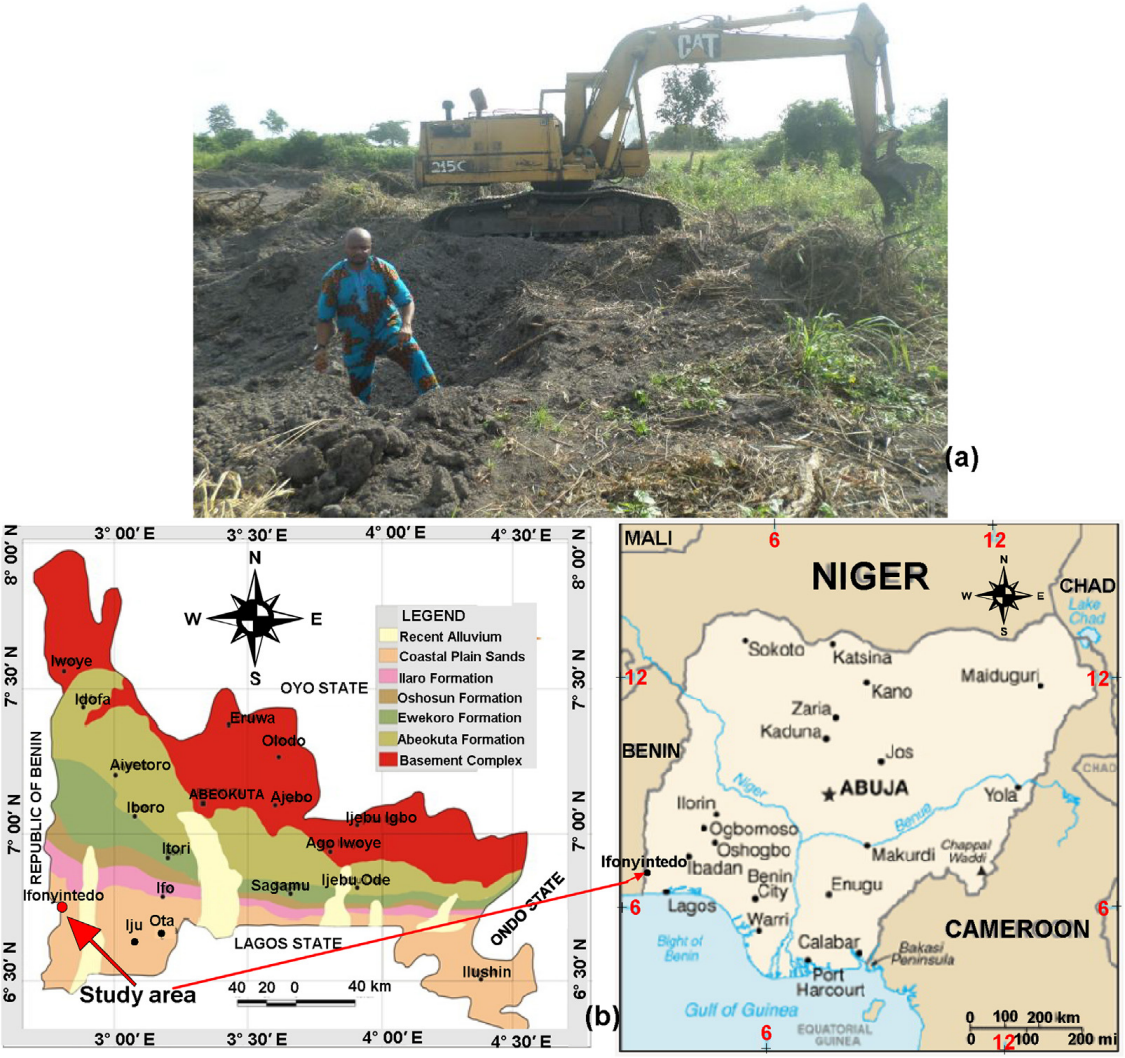
(a) Kaolin mining in Ifonyintedo; (b) Geological domains of Ogun state, Nigeria revealing Ifonyintedo.

Kaolin is one of the mineral resources that are available in commercial quantity in Nigeria. Ifonyintedo, the study area is one of the several locations where kaolin clays are mined in Nigerian sedimentary Basins. The study area is bounded by longitude 002° 47.498′ to 002° 47.570′ E and latitude 006° 46.077′ to 002° 46.126′ N, located in the eastern arm of Dahomey Basin, Nigeria. The elevation above the sea level ranged from 86 to 91 m, with an average of 89 m. Fig. 1a is the representation of how the kaolin clays are mined in Ifonyintedo, Nigeria. Ifonyintedo is a town located in Idiroko local council development area, Ipokia local government area, Ogun state, SW Nigeria. The town has a population of approximately 10, 000. The residents along Ifonyintedo axis are into farming and cottage industry. The major cultivated crops in Ifonyintedo include: cassava, maize, vegetable, and cash crop such as palm tree. The major cottage industries are cassava and palm oil industries. Recently, the discovery of kaolin deposits in commercial quantity has attracted the miners to the town. Commercial activities in Ifonyintedo have been improved greatly, due to its propinquity to the Republic of Benin’s border. Like other suburbs of the study area, Ifonyintedo has a tropical climate, with distinct two seasons: rainy and dry seasons. Averagely, the rainy season span from March to November, while the dry season fluctuates from November to March, except on some minor cases where the rainfall is scarcely experienced between December and January. The mean temperature of the study is 26.5 °C. Additional information on Ifonyintedo is available on [1].

Nigeria is on the Pan-African mobile belt, which separates Congo Cratons and West Africa [16]. In Nigeria, the two pronouncing geology are the Basement rocks and sedimentary Basins. From the literature, few works from both of the terrains could be found in Refs. [[17], [18], [19], [20], [21], [22], [23]]. Ifonyintedo is directly seated on the Eastern region of Dahomey Basin, which is one of the Nigerian Basins. This Basin is situated in SW region of Nigeria, which is separated from the prolific Niger Delta Basin by Okitipupa Ridge. Its depositional constituents are grouped into six classes, these are: Benin, Abeokuta, Oshosun, Akinbo, Ilaro and Ewekoro Formations. The descriptions of these classes have been presented by Adagunodo et al. [1]. The Hydrogeology of Dahomey basin comprises Ogun River and Owena basin. Fig. 1b is the diagrammatic representation of the geological domains in Ogun state revealing the study area.

## Materials and methods

In situ measurements of activity concentrations of K-40, Th-232, U-238 and the gamma dose rates were taken over kaolin deposits in Ifonyintedo, Dahomey Basin, SW Nigeria. The data were randomly occupied at the upper axis of the field from eleven (11) locations using a hand-held detector known as Super-Spec (RS 125). The coordinate and elevation of each location were determined with the aid of global positioning system (GPSMAP 78). The radioactivity measurements were taken four times at each location while their averages and standard deviations were estimated in order to ensure accuracy. All the measured parameters are shown in Table 1. The radiometric survey was carried out in the month of January 2018. The detector used was manufactured by Canadian Geophysical Institute. It has high accuracy with probable measurement errors of about 5%. It offers an integrated design with a large detector, direct assay readout, data storage and high sensitivity. The assay mode of RS-125 Super SPEC provides sample concentration analysis with direct data display of potassium (K) in percentage (%), uranium (U) in part per million (ppm) and thorium (Th) in part per million (ppm). The spectrometer is calibrated on 1 × 1 m test pads, which utilizes 5 min spectra accumulation on potassium, thorium and uranium pads and 10 min accumulation on the Background (BG) pad as calibrated by Canadian Geophysical Institute. It uses sodium iodide (NaI) crystal doped with thallium [Tl] as activator. The approximate linear energy of the detector falls between 0.80 and 1.2 MeV, this range covers the majority of significant gamma-ray emissions from terrestrial sources. The detection of gamma-ray from cosmic ray is negligible due to the detector’s low response to high-energy gamma radiation. The full count of 120 s per assay was adopted for best accuracy as stated in Radiation Solutions Inc. [24]. The recorded activity concentrations of K-40, Th-232, U-238 from the detector were converted to Becquerel per kilogram (Bq kg^−1^) in accordance with the conversion factor of International Atomic Energy Agency [25,26]. The advantage of in situ radiation measurement method over ex situ is that, the measurements are faster; less costly; greater data points can be measured; more than two measurements per station can be achieved, which minimizes the uncertainty on the mean of radioactivity concentrations [27]. Calibration pad for Super-Spec (RS-125) according to Canadian Geophysical Institute is presented in Eqs. (1)–(3).

(1) Q00 – K – pad – % of K = 8.71 for RSI

(2) Q11 – U – pad – eU ppm = 52.9 for RSI

(3) Q22 – Th – pad – eTh ppm = 136.0 for RSI

**Table 1:** Measured concentrations of radionuclides and the absorbed dose rates from the upper axis of kaolin deposits in Ifonyintedo.

Sample	U-238 (Bq/kg)	Th-232 (Bq/kg)	K-40 (Bq/kg)	Dose rate (nGy/hr)	Longitude	Latitude	Elev. (m)
UA1	22.23 ± 0.02	98.25 ± 0.11	125.2 ± 0.90	73.77 ± 1.80	002° 47.516′ E	006° 46.077′ N	87
UA2	18.53 ± 0.02	69.02 ± 0.25	156.5 ± 0.12	55.95 ± 0.65	002° 47.500′ E	006° 46.091′ N	91
UA3	39.52 ± 0.02	60.90 ± 0.30	93.9 ± 0.86	57.63 ± 1.45	002° 47.503′ E	006° 46.095′ N	90
UA4	55.58 ± 0.01	66.18 ± 0.15	31.3 ± 5.40	65.18 ± 2.99	002° 47.498′ E	006° 46.099′ N	88
UA5	48.17 ± 0.02	61.71 ± 0.20	125.2 ± 2.30	63.19 ± 0.65	002° 47.505′ E	006° 46.102′ N	87
UA6	43.23 ± 0.02	58.87 ± 0.15	31.3 ± 0.50	55.41 ± 1.84	002° 47.570′ E	006° 46.109′ N	89
UA7	64.22 ± 0.03	64.55 ± 0.16	62.6 ± 1.49	69.28 ± 0.30	002° 47.513′ E	006° 46.114′ N	88
UA8	43.23 ± 0.02	48.72 ± 0.15	93.9 ± 3.28	51.95 ± 1.56	002° 47.503′ E	006° 46.117′ N	91
UA9	19.76 ± 0.01	62.12 ± 0.29	125.2 ± 1.24	51.04 ± 2.18	002° 47.504′ E	006° 46.120′ N	90
UA10	43.23 ± 0.01	56.03 ± 0.13	125.2 ± 2.58	57.63 ± 0.20	002° 47.514′ E	006° 46.123′ N	89
UA11	22.23 ± 0.01	69.83 ± 0.20	62.6 ± 1.69	54.13 ± 3.63	002° 47.517′ E	006° 46.126′ N	86
Range	18.5 – 64.2	48.7 – 98.3	31.3 – 156.5	51.04–73.77	002° 47.498′ E -002° 47.570′ E	006° 46.077′ N-006° 46.126′ N	86 - 91
Overall mean	38.17	65.11	93.90	59.56	---	---	89

Global average							
[28]	50	50	500	55	---	---	---
[29]	50	50	500	55	---	---	---
[30]	50	50	670	50	---	---	---
[31]	32	45	420	84	---	---	---

## Method descriptions

### Measured radionuclides and gamma dose

The mean and standard deviation of the measured radionuclides (^238^U, ^232^Th and ^40^K) and gamma Dose Rates (DR) per station from the upper axis of kaolin deposits in Ifonyintedo are revealed in Table 1. The highest recorded values for ^238^U, ^232^Th, ^40^K and DR are 64.22 ± 0.03 Bq kg^−1^, 98.25 ± 0.11 Bq kg^−1^, 156.5 ± 0.12 Bq kg^−1^, and 73.77 ± 1.80 nGy h^−1^, respectively, while the least recorded values for the same radionuclides and DR are 18.53 ± 0.02 Bq kg^−1^, 48.72 ± 0.15 Bq kg^−1^, 31.3 ± 0.50 Bq kg^−1^ and 51.04 ± 2.18 nGy h^−1^ respectively. The overall mean values were estimated as 38.17 Bq kg^−1^, 65.11 Bq kg^−1^, 93.90 Bq kg^−1^ and 59.56 nGy h^−1^ in the same order for the radionuclides and DR respectively. The global averages from four standards were compared with the overall mean as revealed in Table 1. The NEA-OECD [28] and UNSCEAR [29] standards revealed that ^238^U and ^40^K were below the community weighted values of 50 and 500 Bq kg^−1^ respectively. The overall mean values of ^232^Th and DR showed that they were above the permissible limits of 50 Bq kg^−1^ and 55 nGy h^−1^ by the factor of 1.3 and 1.1 respectively. The EC [30] standard revealed that the overall mean values of ^238^U and ^40^K were below the community weighted values of 50 and 670 Bq kg^−1^ respectively. For ^232^Th and DR, the overall mean values were above the community weighted values of 50 Bq kg^−1^ and 50 nGy h^−1^ by the factors of 1.3 and 1.2 respectively. The latest standard considered from Table 1 is UNSCEAR [31], which gave the community weighted values for radionuclides (^238^U, ^232^Th and ^40^K) and DR as 32 Bq kg^−1^, 45 Bq kg^−1^, 420 Bq kg^−1^ and 84 nGy h^−1^ respectively. By comparing the community weighted values with the overall mean values in Table 1, it is revealed that ^40^K and DR were below the permissible limit, while ^238^U and ^232^Th were above the community weighted values by the factors of 1.2 and 1.4 respectively. Since the estimated mean values presented in Table 1 are greater than their respective standard deviation values, it indicates that there is high degree of uniformity in the presented data sets [32]. The comparative analysis of the measured radionuclides and DR with some selected studies from literature is revealed in Table 2.

**Table 2:** Comparison of the mean with some selected studies.

Case study	U-238 (Bq/kg)	Th-232 (Bq/kg)	K-40 (Bq/kg)	Dose rate (nGy/hr)	Country	Reference
Phosphogypsum	206.8	99.1	15.1	154.6	Brazil	[33]
Kaolin	964.7	251.6	58.9	581	Egypt	[34]
Lambapur soil	93.1	141.0	---	---	India	[35]
Mallapuram soil	219.0	271.7	---	---	India	[35]
Peddagattu soil	35.8	93.8	---	---	India	[35]
Clay	39.3	49.6	569.5	74.1	Turkey	[36]
Kaolin	82.0	94.8	463.6	117.7	Turkey	[36]
Soil profile 1	13.71	10.45	57.17	15.20	Nigeria	[37]
Soil profile 2	11.49	8.83	59.77	13.30	Nigeria	[37]
Soil	19.16	48.56	1146.88	89.6	India	[38]
Brick, Soil, Cement, Sand and Clay	9.19	45.60	295.11	53.50	India	[32]
Floor ceramic	101.22	87.53	304.57	213.98	Iraq	[39]
Wall ceramic	102.12	70.90	328.60	178.40	Iraq	[39]
Kaolin deposits	38.2	65.1	93.9	59.6	Nigeria	Present study
Soil and rock	32.0	45.0	420.0	84.0	Global	[31]

**Fig. 2:**
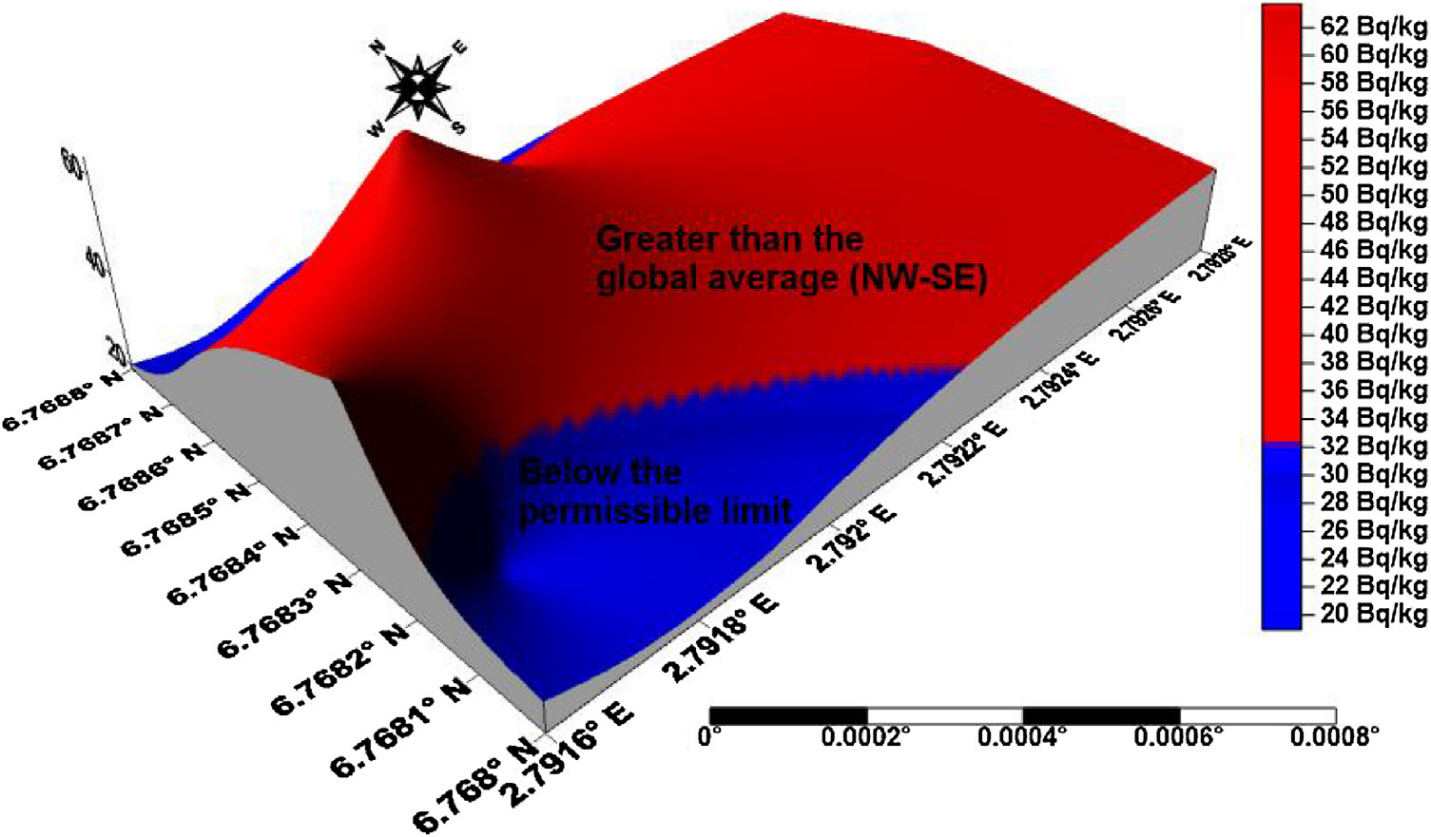
Isouranium map of kaolin deposits in Ifonyintedo (upper axis).

**Fig. 3:**
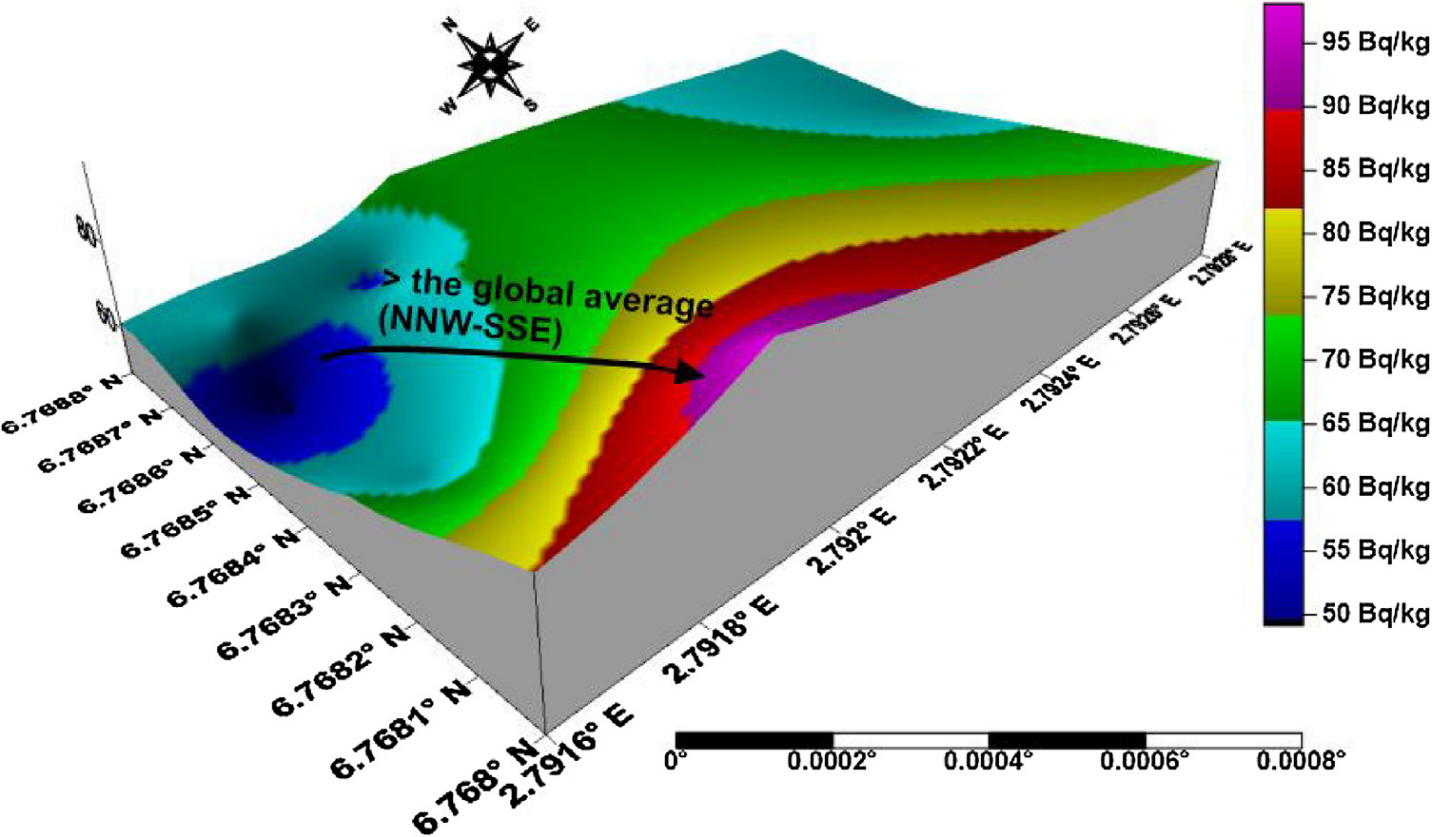
Isothorium map of kaolin deposits in Ifonyintedo (upper axis).

**Fig. 4:**
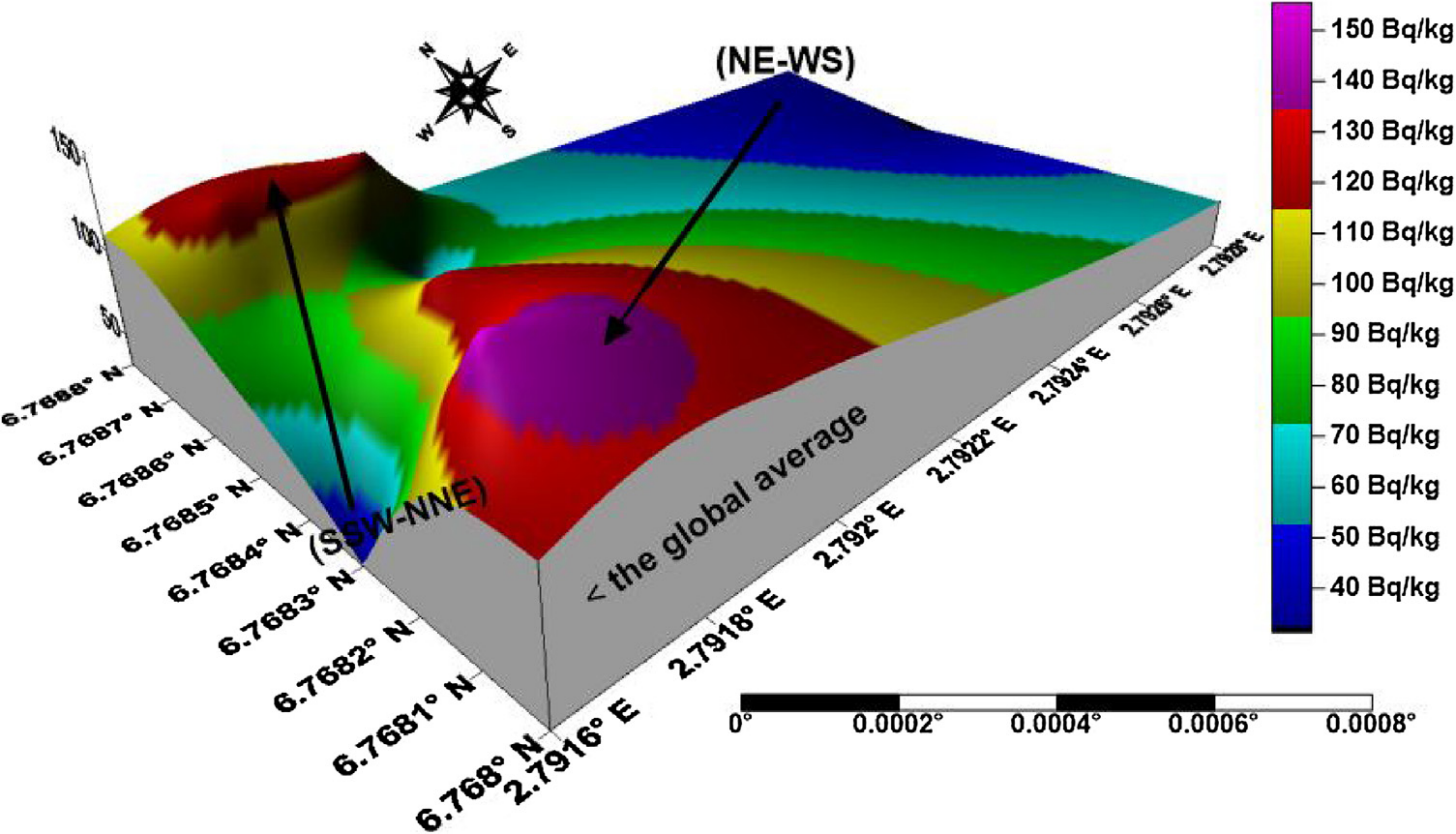
Isopotassium map of kaolin deposits in Ifonyintedo (upper axis).

**Fig. 5:**
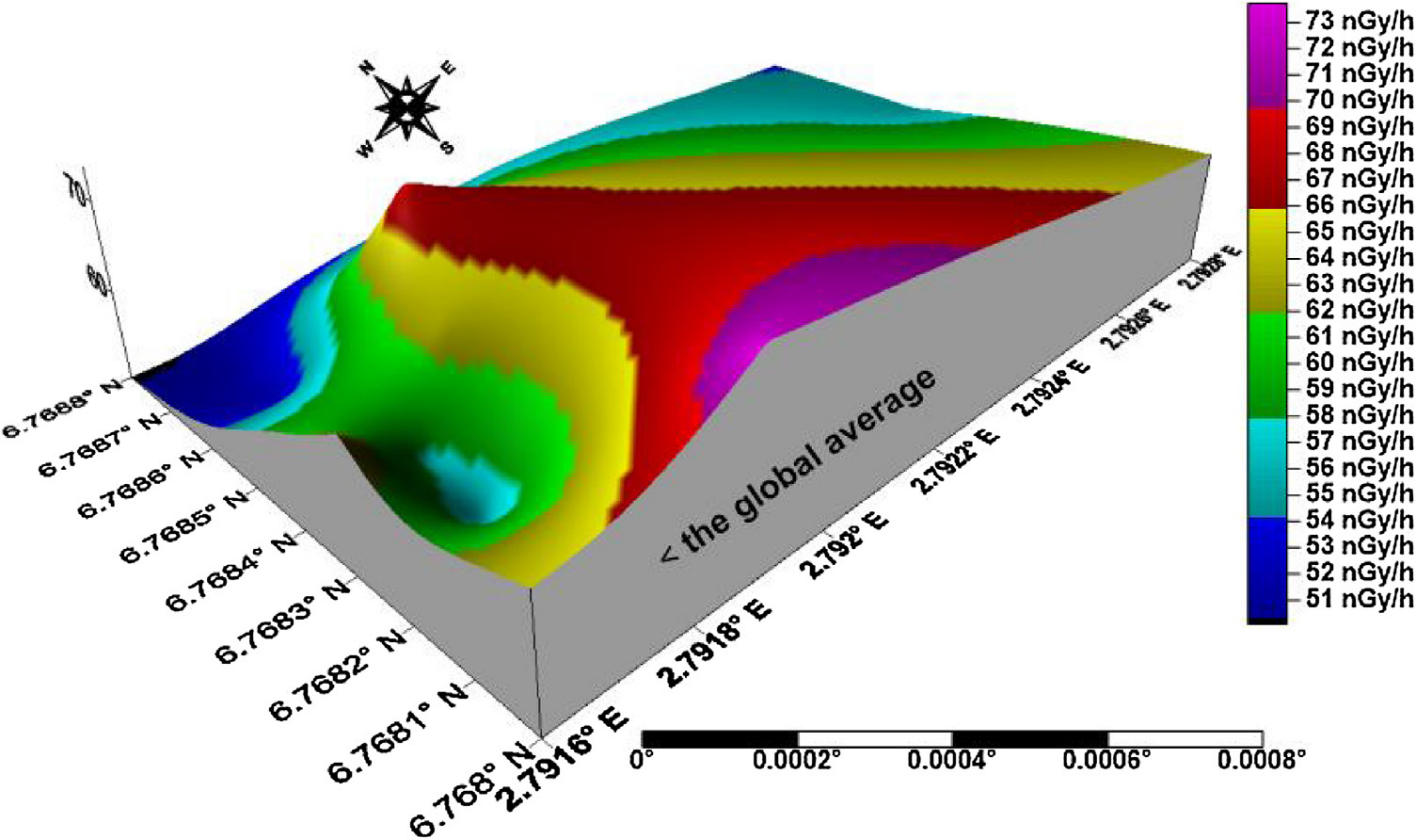
Isodose map of kaolin deposits in Ifonyintedo (upper axis).

The isouranium, isothorium, isopotassium, and isodose maps of kaolin deposits in the upper axis of the field in Ifonyintedo are presented in Figs. 2–5 respectively. Based on the standard set by UNSCEAR [31], the enhanced activity concentrations of uranium are depicted with red colour on Fig. 2. The uranium distributions in the study area trend in NW – SE orientation, with its peak towards the western region. The activity concentrations of thorium trend in NNW – SSE orientation, with its order of increment explained from the colour scale (Fig. 3). Very low potassium activity dominates the study area, which is far lower than the global mean. Nonetheless, two distribution trends were observed from the isopotassium map, which are NE – SW and SSW – NNE orientations (Fig. 4). The gamma isodose map (Fig. 5) revealed that the enhanced activity trend from north to south, and spread towards the SW and SE of the study area respectively. The distributions of the doses are explained from the colour scale of Fig. 5.

**Fig. 6:**
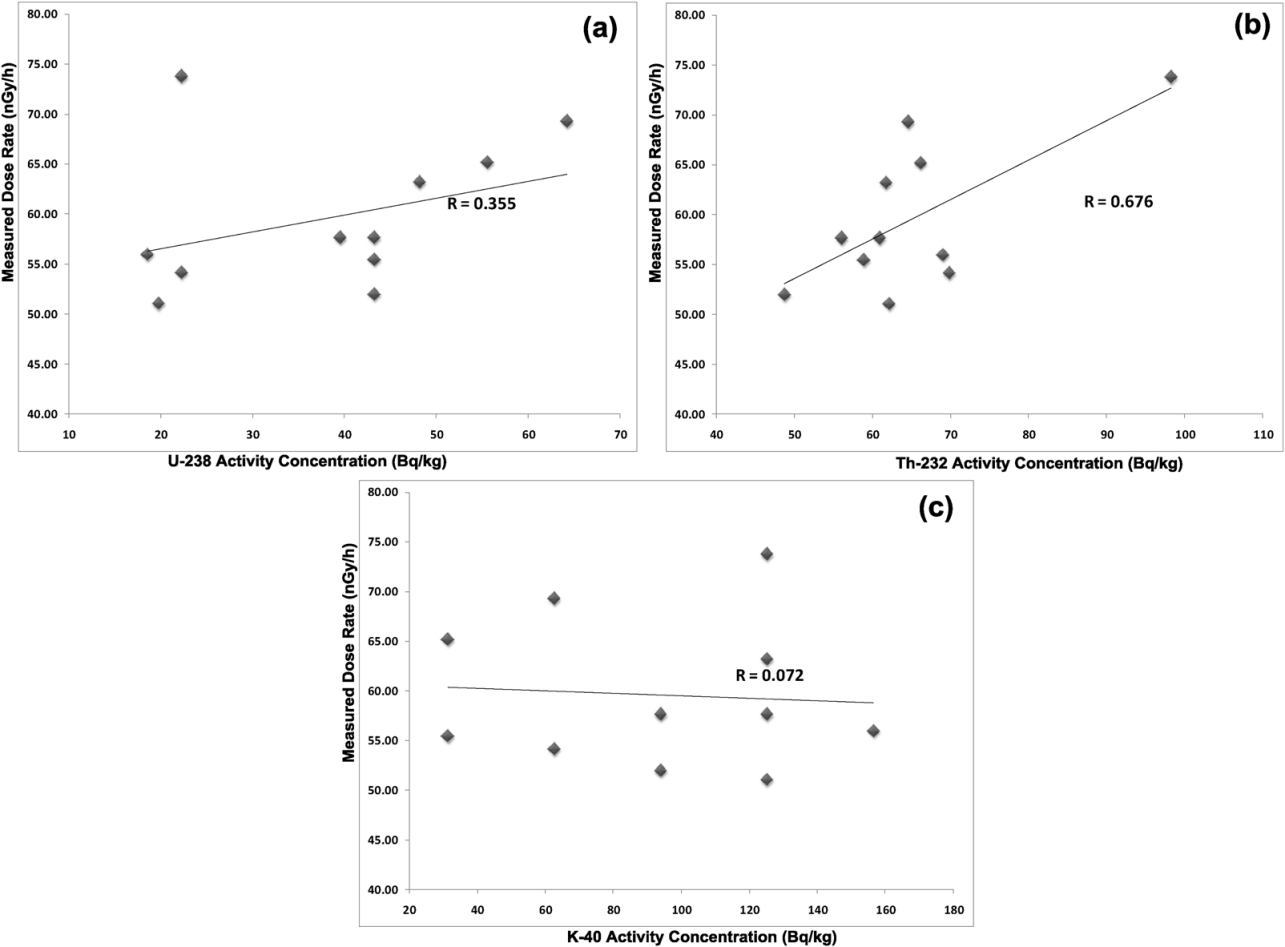
Correlation plots between (a) ^238^U and DR (b) ^232^Th and DR (c) ^40^K and DR.

The correlation studies between the radionuclides and the gamma dose were achieved by plotting the graphs of dose rate against ^238^U (Fig. 6a), dose rate against ^232^Th (Fig. 6b), and dose rate against ^40^K (Fig. 6c) respectively. Correlation study is usually performed between the pairs of radionuclides or / and its gamma dose rate when someone is keen to quick check the relationships that exist between the activity and gamma dose in the area of interest [38]. A weak correlation of 0.355 existed between ^238^U and DR, a fairly good correlation of 0.676 existed between ^232^Th and DR, while a poor correlation of 0.072 existed between ^40^K and DR as revealed in Fig. 6a–c. The correlation results showed that the area of study is enriched in thorium. Hence, the gamma dose received from the kaolin deposits in the upper axis of Ifonyintedo is insignificant as a result of potassium isotopes, but might be weakly significant due to uranium series. Despite the transfer of radionuclides from the raw material to their finished product (such as the case of kaolin to tile), it is imperative to state that the miners on this field need to be aware of the hazards from overexposure to thorium. Thorium is one of radioactive metals that exist in soil, rock, water (surface and ground), and man’s environment. It does not dissolve easily in water, or evaporate to the surface and environs of the Crust. Overexposure to thorium has been linked with cancers of various kind, liver diseases, malfunctioning of the body systems and blood stream related diseases. Generally, overexposure to background radiations has been linked with severe health related problems such as disease of lung, bone, mouth, skin and failure of the body systems, which could result to death in the long term [40].

### Assessment of radiological hazards from kaolin deposits

Eight radiological hazards were determined in order to evaluate the risks that are associated with the mined kaolin deposits in Ifonyintedo as well as the miners. The estimated hazards were radium equivalent, external and internal hazards, outdoor and indoor annual effective doses, gamma and alpha indices, and representative level index. All these estimated hazards are presented in Table 3.

**Table 3:** Summary of the radiological hazards estimate.

ID	Ra_Eq_ (Bq kg^−1^)	H_Ex_	H_In_	AED_Outdoor_ (mSv y^−1^)	AED_Indoor_ (mSv y^−1^)	I_γ_	I_α_	RLI
UA 1	172.37	0.47	0.53	0.36	0.09	0.61	0.11	1.21
UA 2	129.27	0.35	0.40	0.27	0.07	0.46	0.09	0.92
UA 3	133.84	0.36	0.47	0.28	0.07	0.47	0.20	0.94
UA 4	152.62	0.41	0.56	0.32	0.08	0.53	0.28	1.05
UA 5	146.05	0.39	0.52	0.31	0.08	0.51	0.24	1.02
UA 6	129.82	0.35	0.47	0.27	0.07	0.45	0.22	0.90
UA 7	161.35	0.44	0.61	0.34	0.08	0.56	0.32	1.12
UA 8	120.12	0.32	0.44	0.25	0.06	0.42	0.22	0.84
UA 9	118.23	0.32	0.37	0.25	0.06	0.42	0.10	0.84
UA 10	132.99	0.36	0.48	0.28	0.07	0.47	0.22	0.93
UA 11	126.91	0.34	0.40	0.27	0.07	0.44	0.11	0.89
Range	118.23 – 172.37	0.32–0.47	0.37–0.61	0.25–0.36	0.06–0.09	0.42–0.61	0.09–0.32	0.84–1.21
Mean	138.51	0.37	0.48	0.29	0.07	0.48	0.19	0.97
Limit	370.00	<1	<1	0.70	0.05	<1	1	1

#### Radium equivalent

Since the measured activity of ^40^K, ^232^Th and ^238^U are inhomogeneous, it is essential to introduce a common radiological index that evaluates the level of each of the radionuclides in the kaolin deposits. The estimated index, which is known as radium equivalent (Ra_Eq_) is presented in Eq. (4) as presented by Turhan [36].

(4) $$Ra_{Eq}=AC_{U}+\left(\frac{10}{7}\right)AC_{Th}+\left(\frac{10}{130}\right)AC_{K}$$

where AC_U_, AC_Th_ and AC_K_ are the activity concentrations of ^238^U, ^232^Th and ^40^K in Bq kg^−1^ respectively. The implication of Eq. (4) is that the maximum limit of the Ra_Eq_ must not be up to 370 Bq kg^−1^, such that the external dose of less than 1.5 mGy y^−1^ will be maintained [37]. The Ra_Eq_ activity in this study is presented in Table 3. The values ranged between 118.23 and 172.37 Bq kg^−1^, with the mean of 138.51 Bq kg^−1^. Both the range and the mean of Ra_Eq_ values were below the limit of 370 Bq kg^−1^ as reported by UNSCEAR [31].

#### External and internal hazards

Exposure to radiation could be external and/or internal. Eqs. (5) and (6) were used to determined the radiation hazards emanating from the field and the mined kaolin [32].

(5) $$H_{Ex}=0.0027AC_{U}+0.0039AC_{Th}+0.0002A_{K}\leq 1$$

(6) $$H_{In}=0.0054AC_{U}+0.0039AC_{Th}+0.0002A_{K}\leq 1$$

where AC_U_, AC_Th_ and AC_K_ have been defined in Eq. (4). The reduction of the limit of ^238^U to half the numeric value essential to external exposure only is known as the internal hazard index, such that the internal dose received will be <1.5 mSv y^−1^ [39]. The H_Ex_ in this study varied between 0.32 and 0.47, with the mean of 0.37. As reported by Ravisankar [32], the internal exposure to radon and its progeny is managed by H_In_. Therefore, the H_In_ from the kaolin deposits in this study as presented in Table 3 ranged from 0.37 to 0.61, with the overall mean of 0.48. In both cases, the external and internal hazards estimated over a kaolin mining field in Ifonyintedo were less than unity. This implies that the field poses no threat to the miners and the mined kaolin is safe for economic use.

#### Outdoor and indoor annual effective doses

In this present article, outdoor and indoor annual effective doses were estimated from the measured absorbed doses and other factors as reported from literature [37]. In order to estimate the Outdoor Annual Effective Dose (AED_Outdoor_), the dose conversion coefficient (0.7 Sv Gy^−1^) from absorbed dose in air to the effective dose received by the body and outdoor occupancy factor of 0.2 was adopted as given by UNSCEAR [31]. For the Indoor Annual Effective Dose (AED_Indoor_), the occupancy factor of 0.8 was adopted for AED_Indoor_ [37]. This implies 8760 h are in a year. Individuals stayed longer indoor than outdoor in a day, hence the variation in the occupancy factors for outdoor and indoor respectively. Eqs. (7) and (8) were used to estimate the AED_Outdoor_ and AED_Indoor_ respectively [14,29,31,32,37].

(7) AED_Outdoor_ (mSv y^−1^) = Dose rate (nGy h^−1^) × (365 × 24) × 0.2 × 0.7 (Sv Gy^−1^) × 10^−6^

(8) AED_Indoor_ (mSv y^−1^) = Dose rate (nGy h^−1^) × (365 × 24) × 0.8 × 0.7 (Sv Gy^−1^) × 10^−6^

The expected mean annual external effective dose from naturally occurring radionuclides is 0.70 mSv y^−1^, while its internal counterpart is 0.05 mSv y^−1^ [37]. In this study, the AED_Outddor_ ranged from 0.25 to 0.36 mSv y^−1^, while that of AED_Indoor_ ranged from 0.06 to 0.09 respectively. The estimated means for the AED_Outddor_ and the AED_Indoor_ are 0.29 and 0.07 mSv y^−1^ respectively. These results indicate that the study area poses no risk to the miners as well as the materials that will be produced from the kaolin (such as tile).

#### Gamma and alpha indices

Other key hazards that were considered in this study are gamma (I_γ_) and alpha (I_α_) indices respectively. These indices were estimated based on the European Commission [30] standard. Gamma index (I_γ_) is the factor that assesses the γ-radiation hazard(s) associated with the naturally occurring radionuclides in a material. The I_γ_ is determined based on Eq. (9) as given by [28,30].

(9) I_γ_ = 0.3333AC_U_ + 0.0050AC_Th_ + 0.0003AC_K_

Where AC_U_, AC_Th_ and AC_K_ are the same as for other estimated hazards. The permissible range of the outdoor annual effective doses’ contributions to the γ-radiation is 0.3 to 1 mSv y^−1^. Any material or sample that poses the AED_Outdoor_ > this range should be exempted from use as raw materials or finished products [36]. If the I_γ_ ≤ 1, it corresponds to an outdoor dose of 1 mSv y^−1^. However, if the I_γ_ ≤ 0.5, it corresponds to an outdoor dose of 0.3 mSv y^−1^ [14]. From Table 3, the I_γ_ ranged from 0.42 to 0.61, with a mean of 0.48. These results correspond to I_γ_ ≤ 0.5, which gives the outdoor effective dose of 0.3 mSv y^−1^.

The alpha index (I_α_) is used to estimate the exposure to α-radiation associated with radon inhalation from a material. The I_α_ is determined based on Eq. (10) [36].

(10) I_α_ = 0.005AC_U_ (Bq kg^−1^)

where AC_U_ is the activity concentration of uranium in each location. It is measured in Bq kg^−1^. As suggested by the by the European Commission [30], the exhalating radon from a material can be greater than 200 Bq m^−3^ if and only if the activity from uranium concentration is greater than 200 Bq kg^−1^. An I_α_ that is less than or equals 1 corresponds to uranium activity (^238^U) ≤ 200 Bq kg^−1^. The I_α_ as presented in Table 3 ranged from 0.09 to 0.32, with a geometric mean of 0.19. Both the range and the geometric mean results showed that the exposure as a result of α-radiation on the kaolin field is minimal. However, period monitoring is recommended for γ-radiation emanating from the kaolin, since I_γ_ ≤ 0.5, which corresponds to 0.3 mSv y^−1^ of outdoor effective dose that was established on the field.

#### Representative level index

The Representative Level Index (RLI) activity was also determined in this study. The RLI is used to determine the γ-radioactivity level associated with the concentrations of these radionuclides. Eq. (11) was used to estimate the RLI over a kaolin field in Ifonyintedo as proposed by [28] and [38].

(11) $$RLI=\left[\frac{1}{150}\right]AC_{U}+\left[\frac{1}{100}\right]AC_{Th}+\left[\frac{1}{1500}\right]AC_{K}$$

where AC_U_, AC_Th_ and AC_K_ are the activity concentrations of ^238^U, ^232^Th and ^40^K (Bq kg^−1^) respectively. The safety rule is that the RLI < 1 [38,39]. The estimated RLI values are presented in Table 3. The results fluctuated from 0.84 to 1.21, with the overall mean of 0.97. When compared to the limit, it has been revealed that the range is slightly above the limit, while the overall mean ≈1. This implies that the RLI of kaolin deposits in the upper axis of Ifonyintedo field may pose radiation hazard, which might be harmful to the miners and the users of the product(s) from the kaolin deposits if proper periodic monitoring and assessment are neglected on the field.

## Conclusion

For the eleven locations covered in this study, the radiometric measurements of radioactivity concentrations of ^40^K, ^232^Th and ^238^U as well as the gamma doses over a kaolin mining field in Ifonyintedo, Nigeria were achieved using Super-Spec (RS-125) detector. The radiological hazards associated with mining of this mineral deposits and its usability as building material (either as raw material or finished product, as in case of tile) were evaluated. The numbers of conclusions drawn from this study are:

i The range of the estimated mean from four-time in situ measurements per location of ^238^U, ^232^Th, ^40^K and DR spanned from 18.53 ± 0.02–64.22 ± 0.03 Bq kg^−1^, 48.72 ± 0.15–98.25 ± 0.11 Bq kg^−1^, 31.30 ± 0.50–156.50 ± 0.12 Bq kg^−1^, 51.04 ± 2.18–73.77 ± 1.80 nGy h^−1^ respectively. When compared with the recent standard adopted in this study [31], the mean activity concentrations of ^238^U and ^232^Th were above the limit by the factors of 1.2 and 1.4 respectively, while ^40^K and DR were below the limit.
ii The Ra_Eq_ activity ranged from 118.23 to 172.37 Bq kg^−1^ were below the recommended limit of 370 Bq kg^−1^ as given by UNSCEAR [31].
iii The external and internal hazards which ranged from 0.32 to 0.47, and 0.37–0.61 respectively were below the recommended limit of unity as reported by [32] and [39].
iv The outdoor and indoor annual effective doses ranged from 0.25 to 0.36 mSv y^−1^, and 0.06–0.09 mSv y^−1^. The mean of AED_Outdoor_ and AED_Indoor_ were below the limits of 0.70 and 0.05 mSv y^−1^ as presented by UNSCEAR [31] and Avwiri et al. [37].
v The I_γ_ ranged from 0.42 to 0.61, with a mean of 0.48 ≈ 0.5. The gamma index in Ifonyintedo corresponds to I_γ_ ≤ 0.5, which gives the outdoor effective dose of 0.3 mSv y^−1^. The I_α_ ranged from 0.09 to 0.32, with a geometric mean of 0.19. The α-radiation exposures as a result of usage of the mineral deposits as raw material or finished product, or to the miners are minimal, but that of γ-radiation exposure needs periodic monitoring [28, 30,36].
vi The RLI ranged from 0.84 to 1.21, which revealed that some locations (UA1, UA4, UA5 and UA7) are hazardous, because their RLI were beyond the recommended value (i.e. 1.0), while the remaining locations are close to unity as presented in Ravisankar et al. [32] and Chandrasekaran et al. [38]. The overall mean of 0.97 was achieved, which could be approximated to unity.

Ogun state is the leading state producer of solid minerals in Nigeria, with kaolin being one of the major solid minerals mined from the state. It is however recommended that periodic assessment of radiological exposure to the miners and the mined kaolin deposits should be of utmost concern to the Nigerian Environmental Standards and Regulatory Enforcement Agency, since some of the estimated hazards are close or could be approximated to the permissible limit.
